# Structural and kinetic insights into flavin-containing monooxygenase and calponin-homology domains in human MICAL3

**DOI:** 10.1107/S2052252519015409

**Published:** 2020-01-01

**Authors:** Junsoo Kim, Haemin Lee, Yeon Jin Roh, Han-ul Kim, Donghyuk Shin, Sorah Kim, Jonghyeon Son, Aro Lee, Minseo Kim, Junga Park, Seong Yun Hwang, Kyunghwan Kim, Yong Kwon Lee, Hyun Suk Jung, Kwang Yeon Hwang, Byung Cheon Lee

**Affiliations:** a College of Life Sciences and Biotechnology, Korea University, 145 Anam-ro, Seongbuk-gu, Seoul 02841, Republic of Korea; b Biochemistry Laboratory, Department of Biosystems and Biotechnology, Kangwon National University, 1 Kangwondaekak-gil, Chuncheon-si, Gangwon-do 24341, Republic of Korea; c Buchmann Institute for Molecular Life Sciences, Goethe University Frankfurt, Frankfurt am Main, Germany; d Department of Biology, College of Natural Sciences, Chungbuk National University, Cheongju, Chungbuk 361-763, Republic of Korea; e Department of Culinary Art and Food Service Management, Yuhan University, 590 Gyeongin-ro, Bucheon-si, Gyeonggi-do 14780, Republic of Korea

**Keywords:** MICAL, actin depolymerization, calponin-homology domain, F-actin, monooxygenases, structure determination, protein structure, refinement, X-ray crystallography, enzyme mechanisms

## Abstract

MICAL is an oxidoreductase that participates in cytoskeleton reorganization via actin disassembly in the presence of NADPH. Although three MICALs (MICAL1, MICAL2 and MICAL3) have been identified in mammals, only the structure of mouse MICAL1 has been reported. Here, the first crystal structure of human MICAL3, which contains the flavin-containing monooxygenase (FMO) and calponin-homology (CH) domains, is reported. MICAL3 has an FAD/NADP-binding Rossmann-fold domain for mono­oxygenase activity like MICAL1. The FMO and CH domains of both MICAL3 and MICAL1 are highly similar in structure, but superimposition of the two structures shows a different relative position of the CH domain in the asymmetric unit. Based on kinetic analyses, the catalytic efficiency of MICAL3 dramatically increased on adding F-actin only when the CH domain was available. However, this did not occur when two residues, Glu213 and Arg530, were mutated in the FMO and CH domains, respectively. Overall, MICAL3 is structurally highly similar to MICAL1, which suggests that they may adopt the same catalytic mechanism, but the difference in the relative position of the CH domain produces a difference in F-actin substrate specificity.

## Abstract 2

The structure of human MICAL3 provides information about its mechanism.

## 1. Introduction   

Flavin-dependent monooxygenases catalyze a variety of oxygenation reactions, including regioselective, chemoselective and stereoselective oxidation reactions, which can be accomplished by single oxygen transfer to target substrates (Hung *et al.*, 2010 ▸, 2011 ▸). These enzymes are involved in several metabolic processes, including the biosynthesis of hormones and vitamins, the inactivation of signaling molecules, the excretion of xenobiotic substrates and the guidance of axons (Kaya *et al.*, 2015 ▸; Drazic & Winter, 2014 ▸; Lee *et al.*, 2013 ▸; Nadella *et al.*, 2005 ▸). With regard to the regulation of axon guidance, the molecule interacting with CasL (MICAL) protein, which belongs to the flavin-dependent monooxygenase family, has recently received attention for its unique function in controlling actin assembly in conjunction with MsrB1 (Lee *et al.*, 2013 ▸). MICAL is a multi-domain enzyme that is highly conserved across most animal kingdoms and participates in actin cytoskeletal reorganization (Kolk & Pasterkamp, 2007 ▸). It has been functionally characterized to possess stereoselective methionine-oxidation activity for actin disassembly in mice and fruit flies. Its flavin-containing monooxygenase (FMO) domain oxidizes the two conserved methionine residues of actin to methionine-*R*-sulfoxides, while the calponin-homology (CH) domain is involved in actin binding, thereby enhancing the catalytic activity of the FMO domain (Gimona *et al.*, 2002 ▸).

Three MICALs, MICAL1, MICAL2 and MICAL3, have been identified in mammals. They contain FMO and CH domains and share a common function of F-actin disassembly. In addition, MICAL2 and MICAL3 have been reported to localize to the nucleus, but MICAL1 is cytosolic (Lundquist *et al.*, 2014 ▸; Fischer *et al.*, 2005 ▸). Furthermore, MICAL1 is auto-inhibited by its C-terminal coiled-coil domain, but MICAL2 is constitutively active for the regulation of actin stress fibers (Giridharan *et al.*, 2012 ▸). Following the discovery of MICAL in *Drosophila*, its biological role in actin cytoskeleton reorganization has been shown in various organisms. MICALs oxidize two conserved methionine residues of actin to methionine-*R*-sulfoxide in a stereospecific manner in the presence of NADPH, thus leading to the disassembly of F-actin into actin monomers (Giridharan & Caplan, 2014 ▸). MsrB then reduces these methionine-*R*-sulfoxides back to methionines. After this redox change, the actin monomer can be reassembled into an F-actin polymer, thereby conferring cytoskeletal reorganization (Lee *et al.*, 2013 ▸). These cytoskeletal alterations regulate multiple cellular events including cell morphology and axon growth, and exocytosis in various tissues. However, the underlying mechanism of this regulation by MICAL proteins is poorly understood (Wu *et al.*, 2018 ▸). The structure of mouse MICAL1 has been determined, which has helped to provide an understanding of how it functions at the molecular level (Siebold *et al.*, 2005 ▸; Nadella *et al.*, 2005 ▸; Sun *et al.*, 2006 ▸). However, the structures of MICAL2 and MICAL3 have yet to be determined, and thus the lack of structural comparison among mammalian MICALs has limited our understanding of both the shared and unique characteristics of these proteins. Here, we report the first crystal structure of human MICAL3, which contains the FMO and CH domains, at 1.9 Å resolution. Based on a comparison of human MICAL3 with mouse MICAL1 (PDB entry 4txi; Alqassim *et al.*, 2016 ▸), we present their structural similarities and differences, as well as their kinetics, which depend on the interactions of the FMO domain with the CH domain.

## 2. Materials and methods   

### 2.1. Cloning of human MICAL1 and MICAL3   

The DNA regions encoding the FMO and CH domains of MICAL3 (*h*MICAL3_FMOCH_; amino-acid residues 1–700), as well as the FMO domain of MICAL3 (*h*MICAL3_FMO_; amino-acid residues 1–494), were amplified from human cDNA. Truncated forms of MICAL1 comprising the FMO and CH domains (*h*MICAL1_FMOCH_; amino-acid residues 1–616) and the FMO domain (*h*MICAL1_FMO_; amino-acid residues 1–489) were amplified from human MICAL1 cDNA purchased from the Korean Human Gene Bank. The oligonucleotide primers used for amplification can be found in Supplementary Table S1. After PCR amplification, *h*MICAL3_FMOCH_ was cloned into the pET-28b plasmid (Novagen) using the restriction enzymes BamHI and XhoI. Other MICAL forms were digested with the same enzymes and inserted into the pET-28a vector. E213G and R530G substitutions were introduced into *h*MICAL3_FMOCH_ in pET-28b by site-directed mutagenesis (*h*MICAL3_FMOCH_Δ213,530; Haque *et al.*, 2018 ▸). To enhance the solubility of the recombinant MICAL forms, they were transformed and overexpressed in the *Escherichia coli* Rosetta 2 pLysS strain (Novagen). The cells were grown at 37°C to an optical density at 600 nm (OD_600_) of approximately 0.6 in LB medium and were induced at 18°C with 0.5 m*M* isopropyl β-d-1-thiogalactopyranoside (IPTG). After 18 h, the cells were harvested by centrifugation at 2300*g* for 1 h and stored at −20°C.

### 2.2. Protein purification   

For each MICAL form, the harvested cells were lysed by sonication in a buffer consisting of 50 m*M* Tris pH 8.5, 150 m*M* NaCl, 2 m*M* β-mercaptoethanol (Hwang *et al.*, 2018 ▸). After centrifugation at 13 000 rev min^−1^ for 1 h at 4°C, the supernatant was loaded onto an Ni–NTA column (GE Healthcare) equilibrated with binding buffer (50 m*M* Tris pH 8.5, 150 m*M* NaCl, 2 m*M* β-mercaptoethanol) and eluted with elution buffer (50 m*M* Tris, 150 m*M* NaCl, 2 m*M* β-mercaptoethanol, 1 *M* imidazole). The purified MICAL proteins (except for *h*MICAL3_FMOCH_) were concentrated using Amicon ultracentrifugal filters (Ultracel-30, 30 kDa cutoff; Millipore) and stored at −80°C. For the subsequent crystallization experiments, *h*MICAL3_FMOCH_ was concentrated and further purified by gel-filtration chromatography on a Superdex S200 column (GE Healthcare) using elution buffer consisting of 50 m*M* Tris pH 8.5, 100 m*M* NaCl, 1 m*M* 1,4-dithiothreitol, 1% glycerol. The predicted molecular weight of *h*MICAL3_FMOCH_ is ∼79.4 kDa. The monomeric form identified in some fractions during gel-filtration chromatography was collected, concentrated to 25 mg ml^−1^ using Amicon ultracentrifugal filters and stored at −80°C.

### 2.3. Crystallization   

Crystals were grown by sitting-drop vapor-diffusion screening at 293 K using the MCSG-1, MCSG-2, MCSG-3 and MCSG-4 screening kits (Anatrace) on an Intelli-Plate 96 (Hampton Research), in which 0.5 µl human MICAL3 solution was mixed with an equal volume of screening solution. The initial crystallization condition was 0.1 *M* Bicine–NaOH pH 9.0, 10%(*v*/*v*) (±)-2-methyl-2,4-pentanediol (MPD) (Hampton Research). For crystal optimization, we employed the hanging-drop vapor-diffusion method in a 24-well VDX plate (Hampton Research). Crystals were optimized by growth in 0.1 *M* Bicine–NaOH pH 9.2, 7%(*v*/*v*) MPD for one day. For cryoprotection, crystals were transferred into a reservoir solution containing 20%(*v*/*v*) glycerol before flash-cooling in liquid nitrogen.

### 2.4. Structure determination   

A diffraction data set was collected on beamline 5A at Pohang Accelerator Laboratory, Republic of Korea at a wavelength of 0.9794 Å and the data were processed and scaled using *SCALEPACK* and *DENZO* from the *HKL*-2000 program package (Otwinowski & Minor, 1997 ▸). The crystal belonged to space group *P*2_1_, with unit-cell parameters *a* = 65.233, *b* = 94.363, *c* = 71.825 Å. The structure was solved by molecular replacement with mouse MICAL1 (PDB entry 4txi; Alqassim *et al.*, 2016 ▸) and the NMR structure of the CH domain of human MICAL3 (PDB entry 2d88; RIKEN Structural Genomics/Proteomics Initiative, unpublished work) as the initial models. Molecular replacement was performed with *Phenix*, model building was performed with *Coot* and final refinement was conducted with *REFMAC*5 in the *CCP*4 suite (Liebschner *et al.*, 2019 ▸; Emsley *et al.*, 2010 ▸; Winn *et al.*, 2011 ▸).

### 2.5. Electron microscopy   

The purified sample of human MICAL3_FMOCH_ was diluted with 50 m*M* Tris pH 8.5, 100 m*M* NaCl, 1 m*M* 1,4-dithiothreitol, 1% glycerol to a final concentration of 80 n*M*. The treated samples (5 µl) were immediately applied onto a carbon-coated grid which had previously been glow-discharged (Harrick Plasma, Ithaca, New York, USA) for 3 min in air. The grids were negatively stained using 1% uranyl acetate. The prepared grids were examined on a Tecnai 10 transmission electron microscope (FEI, USA) equipped with a lanthanum hexaboride (LaB_6_) cathode operating at 100 kV (the instrumentation used at Kangwon Center for Systems Imaging). Images were collected using a US1000 CCD camera (Gatan, USA) at a magnification of 0.32 nm per pixel (Kim *et al.*, 2012 ▸). Single-particle 3D reconstruction from the 1180 negatively stained individuals was performed using the *EMAN*2 package approach with *C*1 symmetry (Ludtke, 2016 ▸). *UCSF Chimera* was used for the visualization and analysis of 3D volumes (Pettersen *et al.*, 2004 ▸).

### 2.6. Steady-state kinetic measurements   

NADPH consumption was monitored in 96-well microplates at a wavelength of 340 nm using a SpectraMax i3 spectrophotometer (Molecular Devices). All reactions were executed in an F-buffer-based assay mixture. The F-actin was prepared according to the supplier’s instructions. 1 mg of actin from rabbit skeletal muscle (Cytoskeleton Inc.) was reconstituted in 100 µl distilled water. It was diluted to 50 µ*M* in G-buffer (5 m*M* Tris–HCl, 0.2 m*M* CaCl_2_, 0.2 m*M* ATP, 1 m*M* DTT pH 8.0) and incubated for 1 h on ice. Following ultracentrifugation at 100 000g for 1 h at 4°C, the supernatant that contained G-actin was separated carefully. 10× polymerization buffer (50 m*M* Tris–HCl, 500 m*M* KCl, 20 m*M* MgCl_2_, 10 m*M* ATP pH 7.5) was then added to the supernatant and incubated at 25°C for 1 h with shaking. After polymerization, the F-actin was diluted to 8 µ*M* with F-buffer (a mixture of G-buffer and polymerization buffer in a 9:1 ratio) and stored on ice until necessary. NADPH was dissolved in 20 m*M* Tris–HCl to make a 10 m*M* stock that was serially diluted as needed. NADPH and MICAL proteins were prepared at ten times the final concentration and 10 µl of each was added to 80 µl F-buffer (without F-actin) or F-actin. For the NADPH standard, the binding buffer for MICAL purification was added instead of the MICAL protein. All experiments were performed in triplicate, and the reaction was recorded every 10 s for 15 min. Kinetic parameters were calculated by fitting a nonlinear regression to the Michaelis–Menten equation using *GraphPad Prism* 5.

## 3. Results   

### 3.1. Overall structure of human MICAL3_FMOCH_   

**Figure 1:**
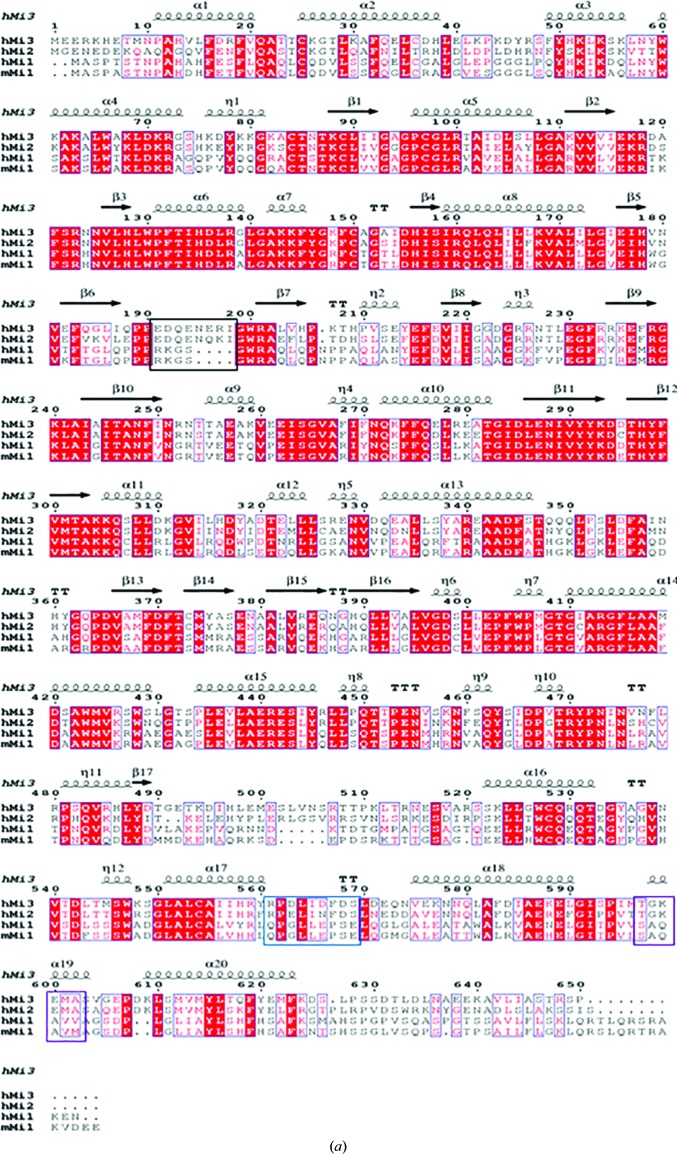

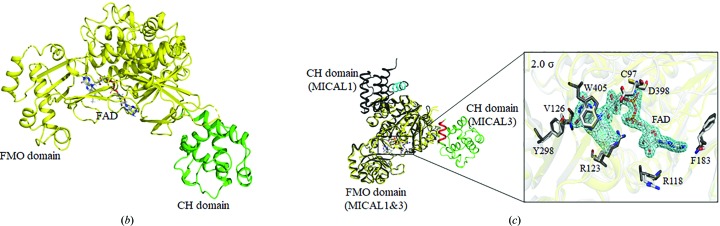
Overall structure and characterization of human MICAL3_FMOCH_. (*a*) Sequence alignment of human MICAL3, human MICAL2, human MICAL1 and mouse MICAL1. Strictly conserved residues are boxed in red, while similar residues are shown as red letters. The sequence-alignment tools used were *ClustalW* and *ESPript*. 3_10_-Helices are represented by η, strict β-turns are represented by **TT** and strict α-turns by **TTT**. (*b*) The crystal structure of human MICAL3. The N- and C-termini are labeled N and C, respectively. The FMO domain is shown in yellow and the CH domain is in green. (*c*) The FAD-binding site. FAD is shown as a stick model and the 2*F*_o_ − *F*_c_ map for the FAD molecule is contoured at 2σ. The distances between residues and FAD were calculated using *PISA*. The stick model of human MICAL3 is shown in yellow, white, red and blue, whereas the ribbon model is shown in yellow; the stick and ribbon models of mouse MICAL1 are shown in black.

To gain structural insights into the role of human MICAL3 (*h*MICAL3) in actin sulfoxidation, recombinant *h*MICAL3 containing the FMO and CH domains (*h*MICAL3_FMOCH_) was expressed and purified for crystallization. The structure of *h*MICAL3_FMOCH_ was determined at 1.9 Å resolution. *h*MICAL3 contains multiple domains: FMO, CH, Lin11, Isl-1, Mec-3 (LIM) and C-terminal coiled-coil domains. In particular, the FMO and CH domains of MICAL3 are homologous to the FMO and CH domains of MICAL1 and MICAL2 [Fig. 1 ▸(*a*)] and are known to play a role in methionine sulfoxidation and actin binding, respectively (Hung *et al.*, 2011 ▸; Giridharan *et al.*, 2012 ▸). The FMO domain of *h*MICAL3_FMOCH_ has the conserved G*X*G*XX*G motif that forms the central part of the consensus *h*MICAL3 sequence connecting the first β-strand and α-helix in the βαβ Rossmann fold. *h*MICAL3 is an oxidoreductase enzyme that depends on FAD and NAD(P) for its catalytic activity (Vanoni *et al.*, 2013 ▸). In the structure, Cys97, Val126, Tyr298, Asp398 and Trp405 interact with one FAD molecule. In particular, the indole ring of Trp405 stabilizes the flavin isoalloxazine ring of the FAD via a coaxial stacking inter­action, while the other residues interact via hydrogen bonds [Figs. 1 ▸(*b*) and 1 ▸(*c*)]. The crystallographic *R*_work_ and *R*_free_ factors are 20.4% and 22.4%, respectively, and the Ramachandran favored region score is ∼98.1% (Table 1 ▸). The final model structure consists of the FMO domain (amino acids 10–491) and the CH domain (amino acids 520–627). These two domains are connected by a flexible loop region (amino acids 492–519). Of the 700 amino acids of *h*MICAL3_FMOCH_, the 73 amino acids at the C-terminus were not observed in the structure. The secondary structure in this region could not be predicted using *JPred* (Cole *et al.*, 2008 ▸), suggesting that this region is flexible.

### 3.2. CH domain of *h*MICAL3_FMOCH_   

**Figure 2:**
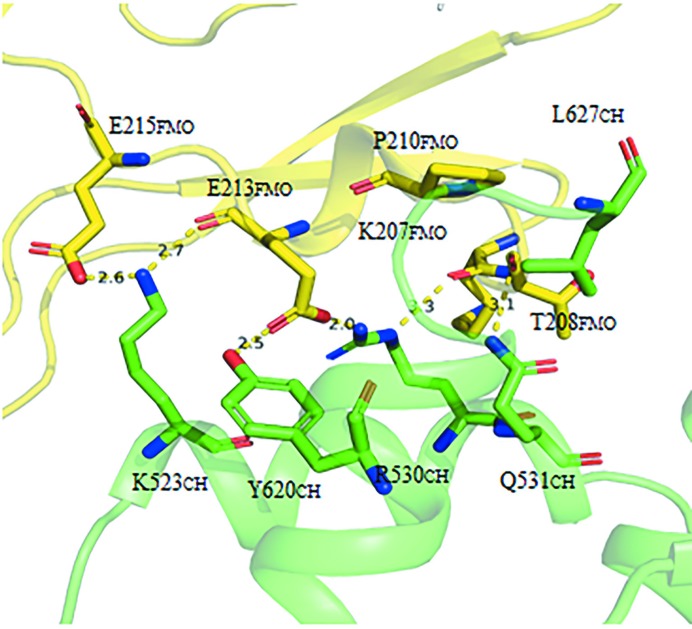
Binding site between the CH domain and the FMO domain in human MICAL3. Residues that participate in the interaction between the FMO and CH domains are labeled. The FMO domain is shown in yellow and the CH domain is shown in green. The binding residues between the FMO domain and the CH domain in human MICAL3 are shown as stick models. The dotted lines indicate the interaction distances between residues.

The CH domain of *h*MICAL3_FMOCH_ is composed of five α-helices and contains a conserved actin-binding motif (522-SKLLGWCQR-530). The *h*MICAL3_FMOCH_ structure shows that the CH domain interacts with the FMO domain, as is the case in mouse MICAL1 (*m*MICAL1; Alqassim *et al.*, 2016 ▸). In particular, five residues, Lys523, Arg530, Gln531, Tyr620 and Leu627, in the CH domain interact with Lys207, Thr208, Pro210, Glu213 and Glu215 in the FMO domain (Fig. 2 ▸). The main chain of Glu213 forms hydrogen bonds to Lys523, and the side chain of Gly213 forms a hydrogen bond and a salt bridge to Tyr620 and Arg530, respectively. In addition, the main chains of Lys207 and Thr208, which correspond to Asn201 and Pro202 of *m*MICAL1, participate in hydrogen bonding to the side chains of Arg530 and Gln531. Interestingly, the other four binding residues of the FMO domain are not conserved in *m*MICAL1, except for Glu215 [Fig. 1 ▸(*a*)].

### 3.3. Interactions between the CH and FMO domains enhance catalytic efficiency   

**Figure 3:**
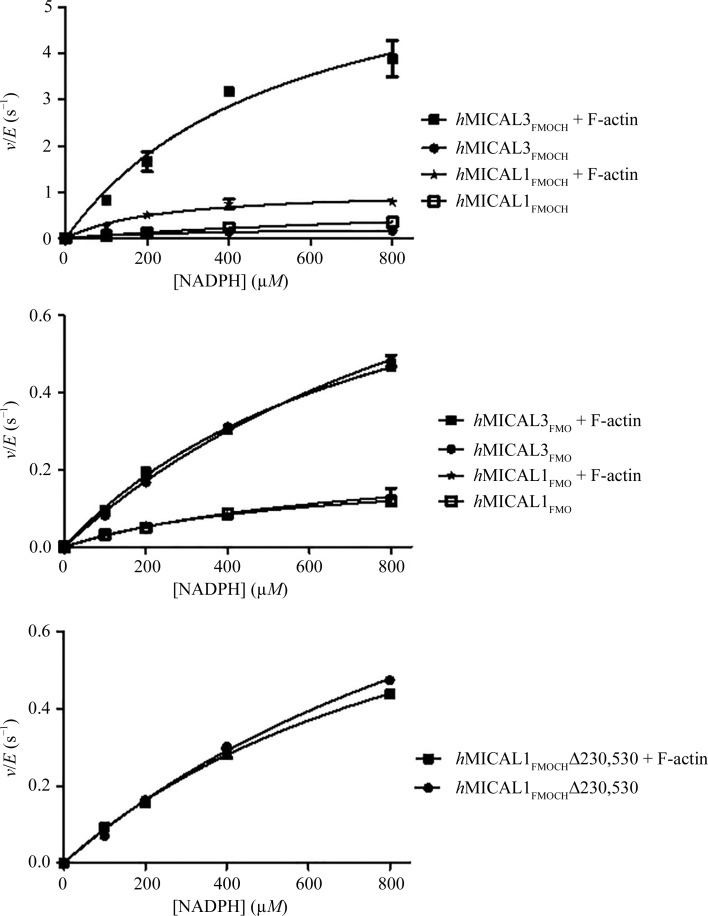
Steady-state kinetic analysis of MICAL forms. Initial velocity (*v*) was measured in an F-buffer-based mixture at various NADPH concentrations. The initial velocity of each reaction was divided by the total enzyme concentration (*E*, 400 n*M*).

Generally, the type 2 CH domain does not bind to F-actin directly. Instead, it facilitates the binding of F-actin to other parts of the protein (Sun *et al.*, 2006 ▸; Gimona & Mital, 1998 ▸; Gimona & Winder, 1998 ▸). To investigate the effects of the CH domain on MICAL enzyme activity, truncated MICAL proteins with or without the CH domain were produced (*h*MICAL1_FMO_, *h*MICAL1_FMOCH_, *h*MICAL3_FMO_ and *h*MICAL3_FMOCH_). It is known that MICAL has NADPH oxidase activity that underlies F-actin disassembly simultaneously with the oxidation of NADPH (Zucchini *et al.*, 2011 ▸). Using this property, kinetic parameters for each MICAL form were determined from the initial velocity of the NADPH oxidase reaction, which depends on the NADPH concentration. The initial velocity of the reaction for *h*MICAL1_FMOCH_ and *h*MICAL3_FMOCH_ at each NADPH concentration was enhanced by adding F-actin, whereas there was no change in the initial velocity for *h*MICAL1_FMO_ and *h*MICAL3_FMO_ when F-actin was added [Figs. 3 ▸(*a*) and 3 ▸(*b*)]. When F-actin was not added, the *k*_cat_/*K*_m_ value for *h*MICAL3_FMO_ (∼2.4794 s^−1^ *M* ^−1^) was similar to that for *h*MICAL3_FMOCH_ (∼2.0776 s^−1^ *M* ^−1^). However, the *k*_cat_/*K*_m_ value for *h*MICAL3_FMOCH_ (∼30.9857 s^−1^ *M* ^−1^) increased dramatically on adding F-actin, while the *k*_cat_/*K*_m_ value for *h*MICAL3_FMO_ (∼2.8793 s^−1^ *M* ^−1^) changed little even after adding F-actin (Table 2 ▸). This increase in catalytic efficiency on adding F-actin when the CH domain is present was also observed in *h*MICAL1. Nevertheless, the ratio of the increase was much higher in *h*MICAL3_FMOCH_ (∼15 times) compared with *h*MICAL1_FMOCH_ (∼6 times). Consequently, these results reveal that the CH domain of *h*MICAL3 participates in increasing the F-actin substrate specificity, leading to enhanced catalytic efficiency. Moreover, it appears that the CH domain of *h*MICAL3 might make a more efficient interaction with the FMO domain for F-actin substrate specificity relative to the CH domain of *h*MICAL1. We chose two residues, Glu213 in the FMO domain and Arg530 in the CH domain, which were predicted to be key residues associated with the interaction between the FMO and CH domains and mutated them to examine the effect of disrupting the FMO–CH interaction on the catalytic efficiency (*h*MICAL3_FMOCH_Δ213,530; E213G and R530G). Notably, the kinetic parameters of *h*MICAL3_FMOCH_Δ213,530, including the initial velocity and catalytic efficiency, are more similar to those of *h*MICAL3_FMO_ than *h*MICAL3_FMOCH_ [Figs. 3 ▸(*b*) and 3 ▸(*c*) and Table 2 ▸]. Moreover, F-actin does not increase the catalytic efficiency of *h*MICAL3_FMOCH_Δ213,530. Therefore, this result suggests that the FMO–CH interaction in *h*MICAL3 is required to increase the catalytic efficiency by conferring specific binding to F-actin.

### 3.4. Structural comparison of CH domains between mouse MICAL1 and human MICAL3   

**Figure 4:**
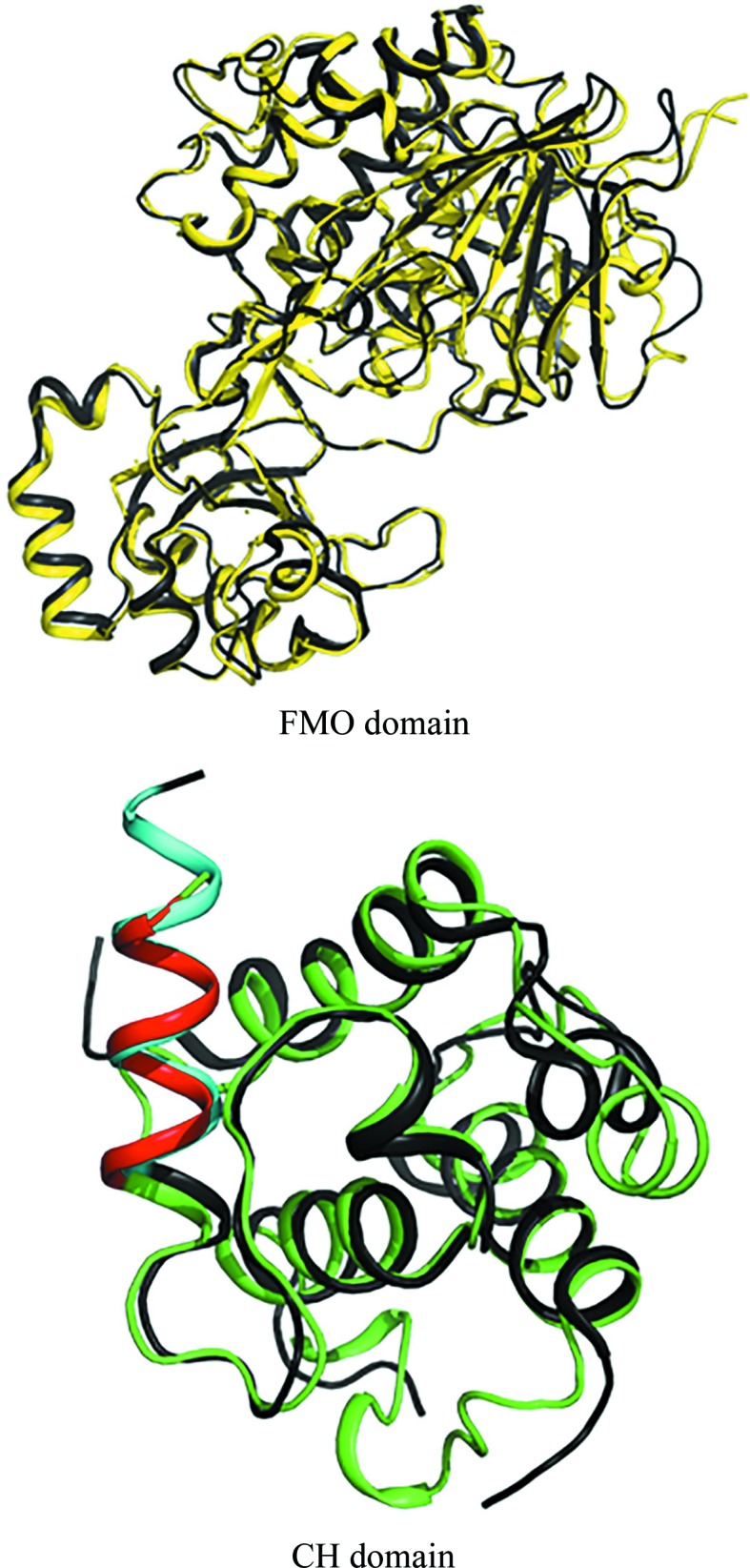
Superimposition of the FMO and CH domains of human MICAL3 and mouse MICAL1. In the upper panel, the FMO domain of human MICAL3 is shown in yellow and the FMO domain of mouse MICAL1 is shown in black. In the lower panel, the CH domain of human MICAL3 is shown in green and the CH domain of mouse MICAL1 is shown in black. The red helix is the actin-binding helix of human MICAL3 and the cyan helix is the actin-binding helix of mouse MICAL1.

**Figure 5:**
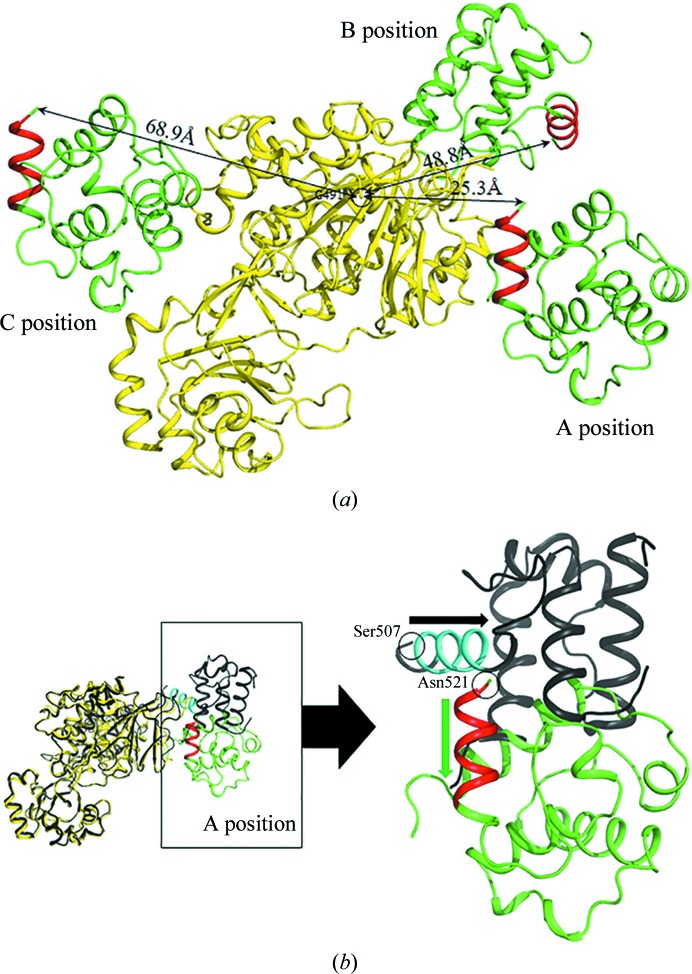
(*a*) Possible orientation of the CH domain in the asymmetric unit. We inferred that the interaction between the FMO and CH domains of human MICAL3 occurs in the A position. Straight lines indicate the shortest distance from the C-terminus of the FMO domain to the N-­terminus of the CH domain. (*b*) Backflip of the CH domain of human MICAL3. Green and red indicate the CH domain and actin-binding helix of human MICAL3, respectively; black and cyan indicate the CH domain and actin-binding helix of mouse MICAL1, respectively. The green arrow indicates the direction from the N-terminus to the C-terminus of the human MICAL3 CH domain; the black arrow indicates the same direction for mouse MICAL1.

**Figure 6:**
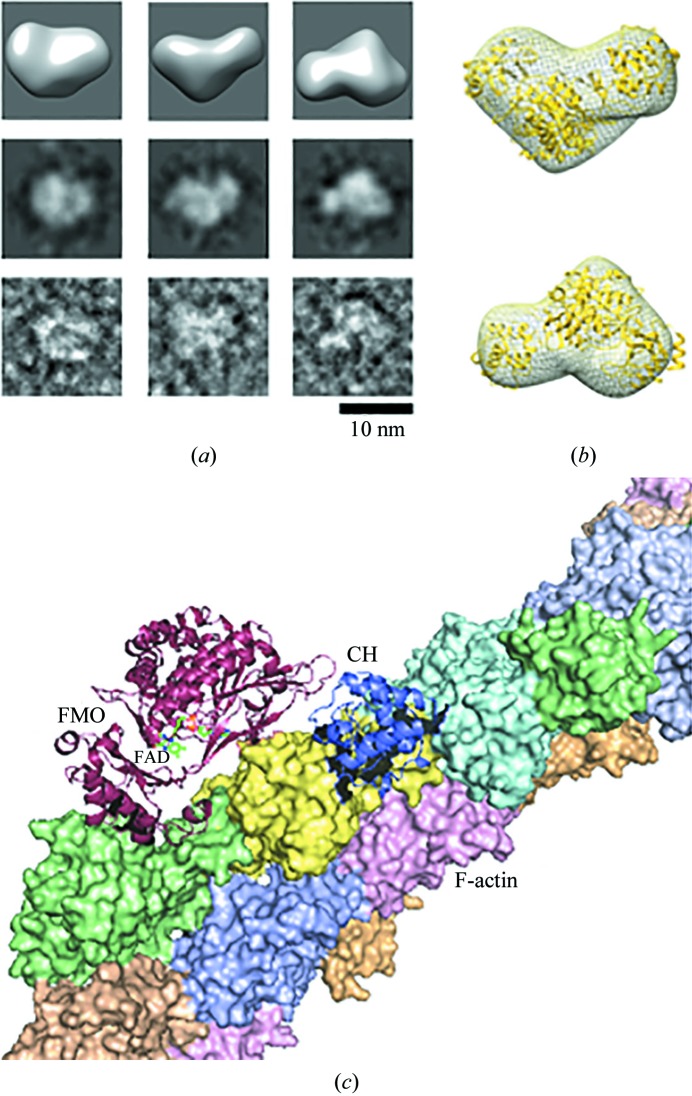
Electron-microscopic analysis of human MICAL3_FMOCH_ and a model of MICAL3_FMOCH_–F-actin interaction. (*a*) Structural comparison taken from 3D analysis: representative surface views of the reconstructed 3D structure (top row) and the corresponding views of 2D class averages (middle row) and raw particles (bottom row). The 10 nm scale bar applies to all of the panels in (*a*). (*b*) Superimposition of an equivalent view of the crystal structure (yellow) on the 3D envelope of negatively stained human MICAL3. (*c*) The initial model was built by manually docking human MICAL3_FMOCH_ to F-actin (PDB entry 3lue; Galkin *et al.*, 2010 ▸). The CH domain (dark blue cartoon) was oriented first and the FMO domain (purple cartoon) was arranged so that the active site was close to actin. Each actin monomer is represented in a different color (tinted surfaces).

The sequence similarity between the FMO domains of *h*MICAL3 (residues 1–494) and *m*MICAL1 (residues 1–489) is ∼58%. The FMO domains showed structural similarity, with a root-mean-square (r.m.s.) deviation of 0.56 Å when 397 C^α^ atoms were aligned in *PyMOL* (Fig. 4 ▸; Janson *et al.*, 2017 ▸). Likewise, the CH domains showed structural similarity, with an r.m.s. deviation of 0.95 Å, and were considered to be type 2 CH domains (Fig. 4 ▸; Janson *et al.*, 2017 ▸). However, the relative position of the CH domain of *h*MICAL3 (*h*MICAL3_CH_) in the crystal structure was different from that in *m*MICAL1. The CH domain can potentially occupy three positions (named positions A, B and C) in the context of an asymmetric unit [Fig. 5 ▸(*a*)]. It was reported that the CH domain of *m*MICAL1 was located at position B (Alqassim *et al.*, 2016 ▸), whereas our structure showed that the CH domain of *h*MICAL3 was located at position A, where it was rotated approximately 90° from position B [Fig. 5 ▸(*b*)]. There is an invisible region (residues 495–517) between the C-terminus of the FMO domain and the N-terminus of the CH domain in the *h*MICAL3_FMOCH_ structure, with a distance of ∼25.3 Å. Essentially, the calculated distances between the FMO and CH domains are ∼25.3 Å for position A, ∼48.8 Å for position B and 68.9 Å for position C. Therefore, considering the distance of the invisible region, the CH domain of *h*MICAL3 fits position A. Furthermore, we performed electron microscopy followed by single-particle analysis to identify the relative position of the CH domain in *h*MICAL3 (Fig. 6 ▸). The visual similarity seen in 3D reconstruction and 2D class averages with corresponding views of raw images [Fig. 6 ▸(*a*)] and the fitting of the crystal structure of MICAL3 to the 3D volume of negatively stained MICAL3 molecules supports the idea that the CH domain is located at position A [*c.f.* the 3D reconstruction in Fig. 6 ▸(*b*) and the models in Fig. 5 ▸(*a*)]. We then built an initial model of the MICAL3_FMOCH_–F-actin interaction [Fig. 6 ▸(*c*)] using position A; the active site seems to be close to the D-loop where the target methionine residues are found.

## 4. Discussion   

MICALs are involved in actin cytoskeleton reorganization through methionine oxidation. However, our understanding of MICALs is limited to genetic and cell biology results, which have been presented in recent papers (Vanoni *et al.*, 2013 ▸; Giridharan & Caplan, 2014 ▸; Lim *et al.*, 2019 ▸). To broaden the scope of these findings, further structural research is required to understand the exact mechanism of how MICALs oxidize methionine. The full-length MICAL protein contains FMO, CH and LIM domains, has a C-terminal domain with unknown function and is highly insoluble, which makes it difficult to obtain a crystal structure. However, its truncated form containing the FMO and CH domains is much more soluble than the full-length form but still retains catalytic activity for F-actin disassembly. Thus, the truncated form of MICAL has been used in various biochemical and biological experiments to gain structural insight into the catalytic mechanism. Of the three mammalian MICALs, only the structure of mouse MICAL1 has been reported and the structure is of a truncated form containing the FMO and CH domains. Interestingly, *m*MICAL1_FMOCH_ and *h*MICAL3_FMOCH_ are structurally highly similar and share the same catalytic function of depolymerizing F-actin via oxidation of the two conserved methionine residues (Fig. 4 ▸). Nevertheless, the crystal structure and electron-microscopy data of *h*MICAL3_FMOCH_ show that the spatial arrangement of the FMO and CH domains differs from that in *m*MICAL1 (Figs. 5 ▸ and 6 ▸). In the crystal structure of *h*MICAL3_FMOCH_, three possible positions of the CH domain could arrange in an asymmetric unit (Fig. 5 ▸) and one of them matches with the observations from electron microscopy. These data also suggest that the crystal structure of *m*MICAL1 differs from the solution shape from SAXS data (Alqassim *et al.*, 2016 ▸).

**Figure 7:**
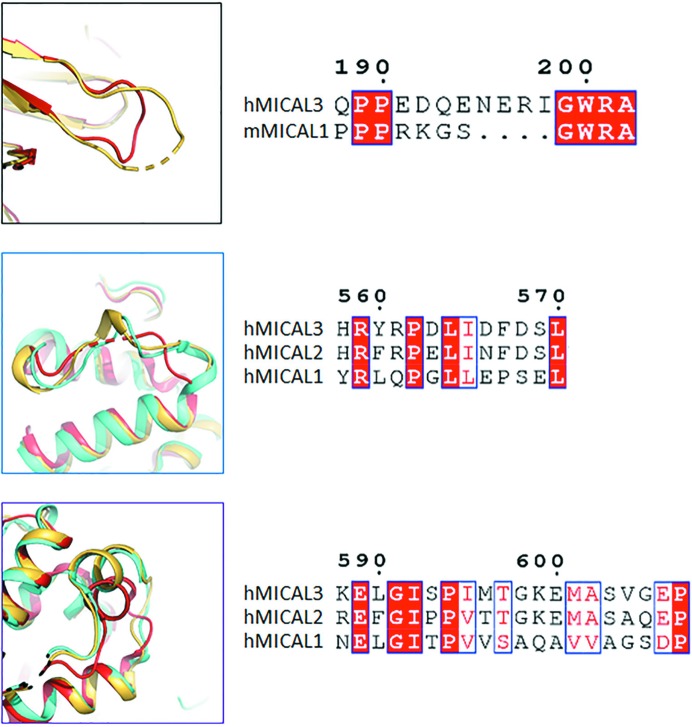
Comparison of the FMO domain and CH domain of MICALs from human and mouse. The sequence alignments between the MICALs are shown on the right for the comparison regions shown on the left. The boxes on the left have the same colors as those highlighting the corresponding sequences in Fig. 1 ▸. In the top left panel, the loop region of the FMO domain of human MICAL3 is in yellow and red indicates the FMO domain of mouse MICAL1 (PDB entry 4txi). The middle and bottom left panels show the superimposition of the CH domain of human MICAL3 in yellow, human MICAL2 (PDB entry 2e9k; RIKEN Structural Genomics/Proteomics Initiative, unpublished work) in cyan and human MICAL1 (PDB entry 2dk9; Sun *et al.*, 2006 ▸) in red.

Both crystal structures clearly show differences, which include the relative location of the CH domain and the length of the invisible region between the FMO and CH domains. The invisible region between the FMO and CH domains is 23 amino acids (495–517) in *h*MICAL3 and 18 amino acids (490–507) in *m*MICAL1. Therefore, this could suggest that different binding conformations are adopted by the FMO and CH domains in *h*MICAL3 and *m*MICAL1. Although *h*MICAL3 and *m*MICAL1 have highly conserved FMO and CH domains (Fig. 3 ▸), there are several reasons why MICAL3 and MICAL1 may have a different mechanism. Firstly, in the FMO domain of *h*MICAL3 the loop is longer than that in *m*MICAL1 and *h*MICAL1 (Fig. 7 ▸). The sequence of this loop is conserved in *h*MICAL3 and *h*MICAL2, but is not conserved in MICAL1. However, the sequence of this loop in MICAL1 is conserved in the human and mouse enzymes (Fig. 7 ▸). Therefore, MICAL2 and MICAL3 may have a similar binding mode by the type 2 CH domain, but MICAL1 does not. *h*MICAL3 and *m*MICAL1 are biologically similar in structure, but it is difficult to determine whether they have the same mechanism (Wu *et al.*, 2018 ▸). In the middle and bottom panels of Fig. 7 ▸, the superimposition of *h*MICAL3, *h*MICAL2 and *m*MICAL1 shows that the α-helices of *h*MICAL2 and hMICAL3 completely superimpose but the α-helix of *m*MICAL1 is only shifted slightly. This sequence also shows that *h*MICAL3 and *h*MICAL2 are completely conserved and *m*MICAL1 is not conserved at all. Therefore, we suggest that *h*MICAL3 and *h*MICAL2 may have a similar mechanism and can be grouped into the same class.

The FMO domain that exhibits monooxygenase activity is localized at the N-terminus of MICAL and is highly conserved among species. The CH domain that is usually found in actin-binding proteins is adjacent to the FMO domain and is also highly conserved. CH domains are classified into three types: types 1, 2 and 3. Whereas type 3 CH domains are mainly found in regulatory proteins associated with muscle contraction and signaling proteins, type 1 and 2 CH domains are usually found in cytoskeletal proteins (Zhou *et al.*, 2011 ▸). MICALs have a typical type 2 CH domain. The results of kinetic experiments with *h*MICAL1 and *h*MICAL3 reveal that when F-actin is present as a substrate, MICAL_FMOCH_ shows a much higher catalytic efficiency than MICAL_FMO_. In the case of MICAL_FMO_, there was no significant change in the activity depending on the presence of F-actin as a substrate. These kinetic data demonstrate that the FMO domain has catalytic activity but that the CH domain must be present for substrate specificity. Moreover, it was shown that the activity of MICAL_FMO_ is slightly higher than that of MICAL_FMOCH_ without F-actin. These two results suggest that the FMO domain performs the catalytic function regardless of the CH domain but that the CH domain is essential for the F-actin substrate specificity of MICALs. In addition, type 2 CH domains do not possess the ability to bind directly to F-actin, as described previously (Vanoni *et al.*, 2013 ▸; Giridharan & Caplan, 2014 ▸; Zhou *et al.*, 2011 ▸). Therefore, we conclude that the interaction between the FMO and CH domains might generate the substrate specificity, particularly for F-actin.

We also examined the change in catalytic efficiency upon mutating the MICAL3_FMOCH_ protein by replacing Glu213 and Arg530, two residues that are important for maintaining the interaction between the FMO and CH domains. Upon mutation, the catalytic efficiency is reduced to levels consistent with *h*MICAL_FMO_. Thus, these findings consistently support the idea that interaction between the FMO and CH domains increases the F-actin substrate specificity and the subsequent catalytic efficiency. Finally, although MICAL has catalytic activity for methionine oxidation, only actin is currently known to be its substrate. The mechanism of sulfoxidation remains disputed and is considered to be owing to direct oxidation by the transfer of a single oxygen molecule or via an indirect oxidation by the production of reactive oxygen species such as hydrogen peroxide. However, it was found that consumption of NADPH without F-actin substrate produced hydrogen peroxide in MICAL1 and MICAL3, but this was not proportional to the activity level of F-actin disassembly (Wu *et al.*, 2018 ▸). Consequently, these findings suggest that the CH domain is crucial for conferring F-actin disassembly via methionine oxidation. In this context, the CH domain of MICAL3 interacts with the FMO domain in more efficient ways than the CH domain of MICAL1.1

## Supplementary Material

PDB reference: human MICAL3, 6ici

Supplementary table and figures. DOI: 10.1107/S2052252519015409/lz5030sup1.pdf

## Figures and Tables

**Table 1:** Data-collection and refinement statistics for *h*MICAL3_FMOCH_ Values in parentheses are for the highest resolution shell.

Data collection	
Wavelength (Å)	0.9794
Space group	*P*2_1_
*a*, *b*, *c* (Å)	65.211, 93.466, 71.568
α, β, γ (°)	90, 92.366, 90
Resolution range (Å)	47.2–2.30 (2.38–2.30)
Completeness (%)	99.7 (99.8)
*R*_sym_ † (%)	0.143 (0.48)
*I*/σ(*I*)	16.89 (2.72)
Redundancy	4.85 (4.8)
Total reflections	38165
Refinement statistics	
Resolution range (Å)	47.2–2.30 (2.38–2.30)
Unique reflections	3817
*R*_work_ (%)	17.2 (19.6)
*R*_free_ (%)	21.5 (24.6)
R.m.s. deviations	
Bond lengths (Å)	0.009
Bond angles (°)	0.097
Ramachandran favored (%)	97.9
Ramachandran outliers (%)	0

† *R*_sym_ = 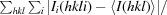 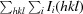, where *I*_*i*_(*hkl*) is the observed intensity of reflection *i*, 〈*I*(*hkl*)〉 is the average intensity and *i* counts through all symmetry-related reflections.

**Table 2:** Steady-state kinetic parameters of various MICALs

Protein	F-actin	*k*_cat_ (s^−1^)	*K*_m_ (µ*M*)	*k*_cat_/*K*_m_ (s^−1^ *M* ^−1^)
*h*MICAL3_FMOCH_	—	0.0006	266.9	2.0776
	8 µ*M*	0.0169	543.8	30.9857
*h*MICAL1_FMOCH_	—	0.0023	1237	1.8238
	8 µ*M*	0.0027	234.1	11.4267
*h*MICAL3_FMO_	—	0.0031	1264	2.4794
	8 µ*M*	0.0024	817.2	2.8793
*h*MICAL1_FMO_	—	0.0005	573.7	0.8911
	8 µ*M*	0.0006	752.6	0.8330
*h*MICAL3_FMOCH_Δ213,530	—	0.0033	1438	2.3282
	8 µ*M*	0.0025	1053	2.4169
