# Supplementary material for: Structural and kinetic insights into flavin-containing monooxygenase and calponin-homology domains in human MICAL3
Source: IUCrJ. 2020 Jan 1;7(Pt 1):90–9. doi: 10.1107/S2052252519015409 (PMC6949599; doi:10.1107/S2052252519015409)
Supplement: Supplementary file 1 [file m-07-00090-sup1.pdf]

IUCrJ

Volume 7 (2020)

Supporting information for article:

Structural and kinetic insights into flavin-containing monooxygenase and calponin-homology domains in human MICAL3

Junsoo Kim, Haemin Lee, Yeon Jin Roh, Han-ul Kim, Donghyuk Shin, Sorah Kim, Jonghyeon Son, Aro Lee, Minseo Kim, Junga Park, Seong Yun Hwang, Kyunghwan Kim, Yong Kwon Lee, Hyun Suk Jung, Kwang Yeon Hwang and Byung Cheon Lee

Table S1 Oligonucleotide primers used for amplification of DNA fragments of MICAL forms.

Restriction enzyme sites are indicated as capitalized (BamH1) and bold (Xho1). Each MICAL form was amplified by primers as follows: *h*MICAL1$_{\mathrm{FMOCH}}$ (1, 2), *h*MICAL1$_{\mathrm{FMO}}$ (1, 3), *h*MICAL3$_{\mathrm{FMOCH}}$ (4, 5) and *h*MICAL3$_{\mathrm{FMO}}$ (4, 6).

	Oligonucleotide	Sequence (5′→3′)
1	MICAL1 Fw	aaGGATCCatggcttcacctacctccaccaa
2	MICAL1 FMOCH Rv	aa**ctcgag**ctagtgggccatgctcttgaaggca
3	MICAL1 FMO Rv	aa**ctcgag**ctactccttggctagcacatcatacaggt
4	MICAL3 Fw	aaGGATCCatggaggagaggaagcatgagacca
5	MICAL3 FMOCH Rv	aa**ctcgag**ctatctttctcctctgtggccccga
6	MICAL3 FMO Rv	aa**ctcgag**ctattttgtttcgccagtatcatataaatggcgca

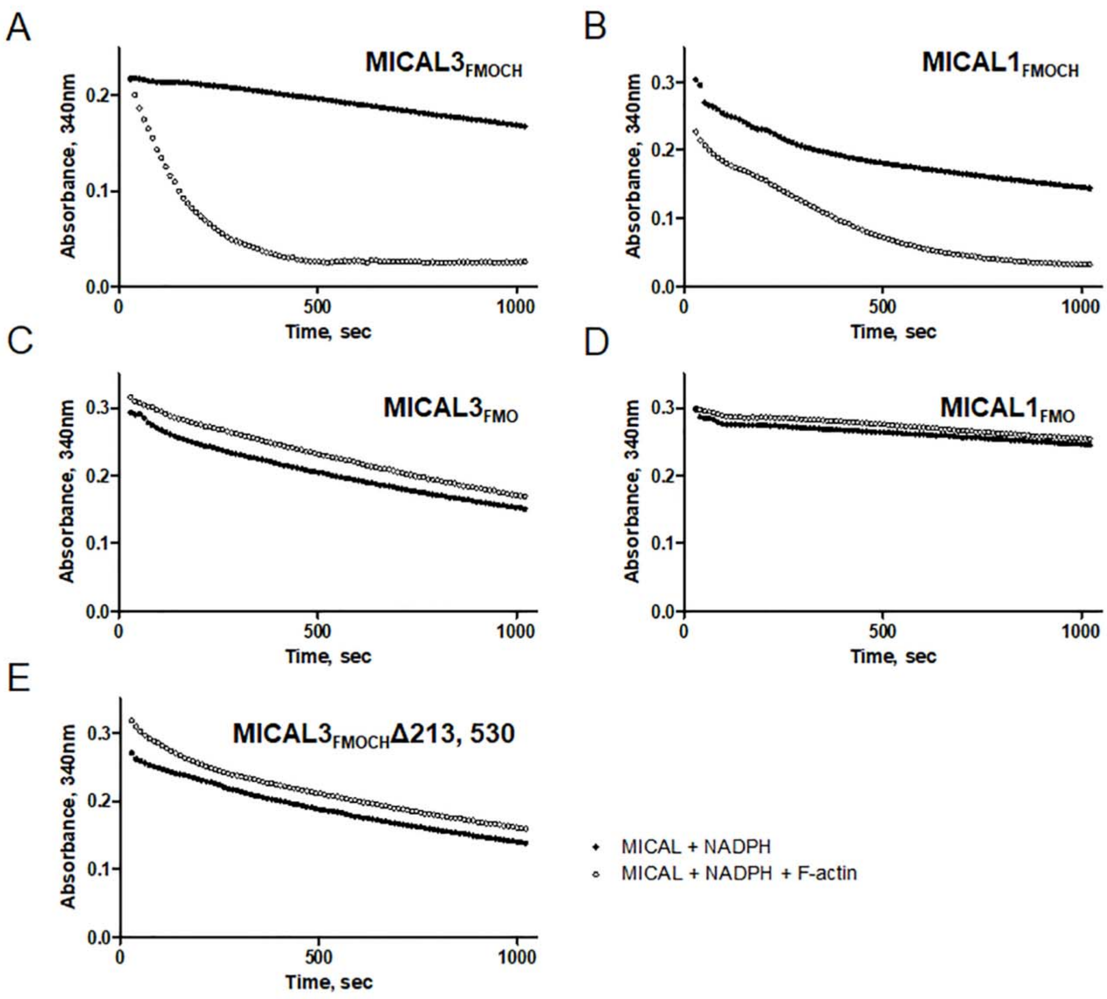

Figure S1 Time-course of NADPH oxidation catalyzed by MICAL forms. The reaction of NADPH oxidation catalyzed by each MICAL form (600nM) was monitored at 340 nm for 15 min. The initial concentration of NADPH was 200 μM and the reaction was progressed with or without F-actin (8 μM).

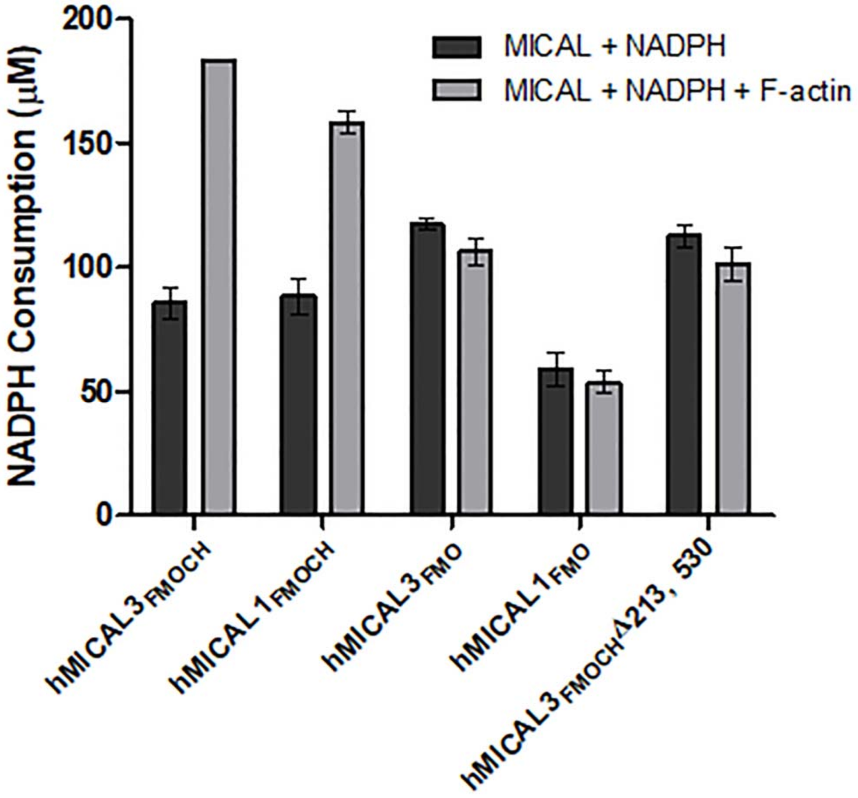

Figure S2 Total NADPH consumption in 15 min. Each MICAL form (600 nM) was incubated with 200 μM of NADPH in the absence (black bar) or in the presence (gray bar) of F-actin (8 μM). NADPH consumption was calculated by subtracting the final NADPH concentration from the NADPH standard.
